# The Utility of Oncology Information Systems for Prognostic Modelling in Head and Neck Cancer

**DOI:** 10.1007/s10916-023-01907-6

**Published:** 2023-01-14

**Authors:** Damian P. Kotevski, Robert I. Smee, Matthew Field, Kathryn Broadley, Claire M. Vajdic

**Affiliations:** 1 Department of Radiation Oncology, Prince of Wales Hospital, Level 1, Bright Building, Barker St, Randwick, NSW 2031 Australia; 2 Prince of Wales Clinical School, Faculty of Medicine, University of New South Wales, Kensington, NSW Australia; 3 Department of Radiation Oncology, Tamworth Base Hospital, Tamworth, NSW Australia; 4 South Western Sydney Clinical School, Faculty of Medicine, University of New South Wales, Kensington, NSW Australia; 5 Ingham Institute for Applied Medical Research, Liverpool, NSW Australia; 6 Cancer and Haematology Services, Prince of Wales Hospital, Randwick, NSW Australia; 7 Centre for Big Data Research in Health, Faculty of Medicine, University of New South Wales, Kensington, NSW Australia; 8 Kirby Institute, Faculty of Medicine, University of New South Wales, Kensington, NSW Australia

**Keywords:** Head and neck cancer, Electronic health records, Oncology information systems, Data quality, Radiation oncology

## Abstract

Cancer centres rely on electronic information in oncology information systems (OIS) to guide patient care. We investigated the completeness and accuracy of routinely collected head and neck cancer (HNC) data sourced from an OIS for suitability in prognostic modelling and other research. Three hundred and fifty-three adults diagnosed from 2000 to 2017 with head and neck squamous cell carcinoma, treated with radiotherapy, were eligible. Thirteen clinically relevant variables in HNC prognosis were extracted from a single-centre OIS and compared to that compiled separately in a research dataset. These two datasets were compared for agreement using Cohen’s kappa coefficient for categorical variables, and intraclass correlation coefficients for continuous variables. Research data was 96% complete compared to 84% for OIS data. Agreement was perfect for gender (κ = 1.000), high for age (κ = 0.993), site (κ = 0.992), T (κ = 0.851) and N (κ = 0.812) stage, radiotherapy dose (κ = 0.889), fractions (κ = 0.856), and duration (κ = 0.818), and chemotherapy treatment (κ = 0.871), substantial for overall stage (κ = 0.791) and vital status (κ = 0.689), moderate for grade (κ = 0.547), and poor for performance status (κ = 0.110). Thirty-one other variables were poorly captured and could not be statistically compared. Documentation of clinical information within the OIS for HNC patients is routine practice; however, OIS data was less correct and complete than data collected for research purposes. Substandard collection of routine data may hinder advancements in patient care. Improved data entry, integration with clinical activities and workflows, system usability, data dictionaries, and training are necessary for OIS data to generate robust research. Data mining from clinical documents may supplement structured data collection.

## Introduction

Globally, head and neck cancer (HNC) contributed to 4.9% of all new cancers and 4.7% of all cancer deaths in 2020 [1]. Contrastingly, within New South Wales (NSW), Australia, 2.8% and 2.7% of all new projected cases and deaths respectively in 2022 will be due to HNC, with a projected increase in incidence and death by 15.8% and 17.3% respectively since 2017 [2]. Predicting survival outcomes in patients with HNC using robust data can help guide treatment practices to improve care and outcomes.

In NSW, cancer data can be accessed from the Australian Cancer Database [3], or the NSW Central Cancer Registry [4], however, neither resource includes the clinical detail necessary to predict HNC survival. Unavailable prognostic factors include smoking history [5], comorbidities [6], human papillomavirus infection [5, 7], fitness for surgery [8], cancer operability [8], size and location of involved lymph nodes in the neck [5, 9, 10], and radiotherapy dose and duration [5, 8, 11, 12].

As part of routine clinical practice, HNC data are entered into structured or free-text fields within the oncology information system (OIS), extracted using structured query language [13], and reported to the NSW Central Cancer Registry. Independently, research personnel at Prince of Wales Hospital (POWH), in Sydney Australia, have compiled a HNC dataset for research purposes using information sourced from clinical documents uploaded into the OIS. The documents are reports from clinical, pathological, and imaging examinations; treatment summaries; correspondence; discharge notes; and follow-up consultations. High quality HNC research datasets are scarce; therefore, the suitability of utilising routinely collected OIS data for outcomes analysis is warranted. Conversely, real-world oncology data is used extensively for other purposes [14], including billing and reimbursement for healthcare services [15, 16], documentation, assessment, and provision of clinical care and treatment pathways [17], epidemiology of disease incidence, prevalence, and trends for disease monitoring [18], clinical trial drug development [19], and machine learning (clinical decision-making, treatment planning, image segmentation, and image guidance) [20].

To provide insight into the utility of OIS, previous studies have investigated data quality and completeness. One study compared the utilisation of the same OIS by two hospitals in Australia, finding one used the full capacity of the OIS fields, while the other focused predominantly on booking and patient tracking [21]. A second study compared the concordance of clinical data in cancer patients between an OIS and a cancer registry. Smoking was highly complete in the OIS; however, only moderate agreement was evident [22]. A third study investigated whether cancer quality measures from an OIS were adequate for Medicare reimbursement in the US [23]. The study reported varying data completion, consequent of underfilling and inconsistencies in OIS data elements, and concluded automated OIS extracts could not yet replace manual abstraction. This study investigates the quality and completeness of OIS data for prognostic modelling in HNC at a major metropolitan teaching and tertiary referral hospital.

## Methods

### Patient eligibility criteria

Adult patients aged 18 years or older presenting at POWH between 1 January 2000 and 31 December 2017 with a newly diagnosed invasive or in-situ mucosal HNC of squamous cell carcinoma morphology, treatment with definitive radiotherapy (± chemotherapy or surgery), known stage, and nil distant metastases, were eligible. Patients diagnosed with cancer of the external lip or commissure, or distant metastases, were excluded. The 2009 Union for International Cancer Control TNM 7th edition manual was used to clinically stage all patients in this study.

### Data collection

The research dataset was previously compiled by trained research personnel. Demographic, diagnostic, treatment, and outcome data was extracted from clinical, histopathological, treatment, and follow-up documents to create a structured dataset (SESLHD HREC 10/040). The research dataset is considered the gold standard dataset for this study. Death data for the research dataset was sourced from: the National Death Index (NDI) via probabilistic record linkage (EO2017/5/392) in February 2018; internal hospital records; the NSW Registry of Births, Death and Marriages; and the Ryerson Index (death notices and obituaries in Australian newspapers). Due to lags adjudication, cause of death data was unavailable for patients registered on the NDI in the two years prior to linkage (2016–2017).

The OIS dataset consisted of routinely collected HNC data extracted from the POWH OIS (MOSAIQ, version 2.60, by Elekta [24]), a proprietary electronic medical record, using structured query language (SQL Server 8 – Crystal Reports). The OIS contains fields for administrative information (e.g., personal details and appointments), patient characteristics (e.g., tobacco use, performance score), disease features (e.g., ICD codes, diagnosis date, staging, histology, morphology), treatment details (e.g., radiotherapy, chemotherapy, surgery, hormone/immunotherapy), and follow-up (e.g., disease and vital status). The OIS is a stand-alone system used by radiation oncology departments in Australia with consistency in the availability of the OIS fields required for mandatory government reporting [25, 26].

Radiotherapy and chemotherapy data in the OIS dataset required manual curation post-extraction. Data extraction resulted in multiple records for each patient, one for each anatomical site receiving radiotherapy. Patients could receive radiotherapy to local, regional, or distant sites, with variation in the terms used to define the radiotherapy treatment sites. Duplicates were removed based on fields uniquely identifying the delivered doses. Radiotherapy data for each diagnosis was manually reviewed by a radiation oncologist to determine the correct dose/fractions to the primary site and neck, which were combined to provide a single value for dose and fraction. Radiotherapy duration was defined as the number of days between the first and last fraction. For quality assurance, radiotherapy data was validated against the full medical record on a random sample of 30 patients.

### Statistical analysis

Forty-four demographic, tumour, treatment, and outcome variables of clinical relevance in HNC survival were analysed. Data was reported as frequencies (%) for categorical data and mean (standard deviation) and median (interquartile range, IQR) for normally and non-normally distributed continuous data respectively (distribution determined using the Shapiro-Wilk test).

The McNemar test was used to determine differences in the distribution of categorical values between the two datasets. The McNemar-Bowker test was used for data with more than two levels. A paired t-test was used to assess differences between normally distributed continuous data, while the Wilcoxon Signed Rank test was used for non-normally distributed continuous data.

Cohen’s kappa coefficient (κ) was used to investigate agreement between categorical variables, reported alongside the standard error (SE). We adapted the Landis et al. [27] interpretation of κ, with 0 to < 0.2 classified as poor agreement, 0.2 to < 0.4 as slight, 0.4 to < 0.6 as moderate, 0.6 to < 0.8 as substantial, 0.8 to < 1.0 as high, and 1.0 as perfect. Intraclass correlation coefficients with two-way mixed models assessing absolute agreement were used to investigate agreement between continuous variables, reported alongside a 95% confidence interval (95% CI).

The level of significance for all tests was *P* < 0.05 and all *P* values are two-sided. Statistical analysis was performed using SPSS 26 (IBM, Armonk, New York), and all analysis was paired.

### Ethical considerations

The study was approved by the NSW Population and Health Services Research Ethics Committee (2019/ETH12196).

## Results

**Fig. 1:**
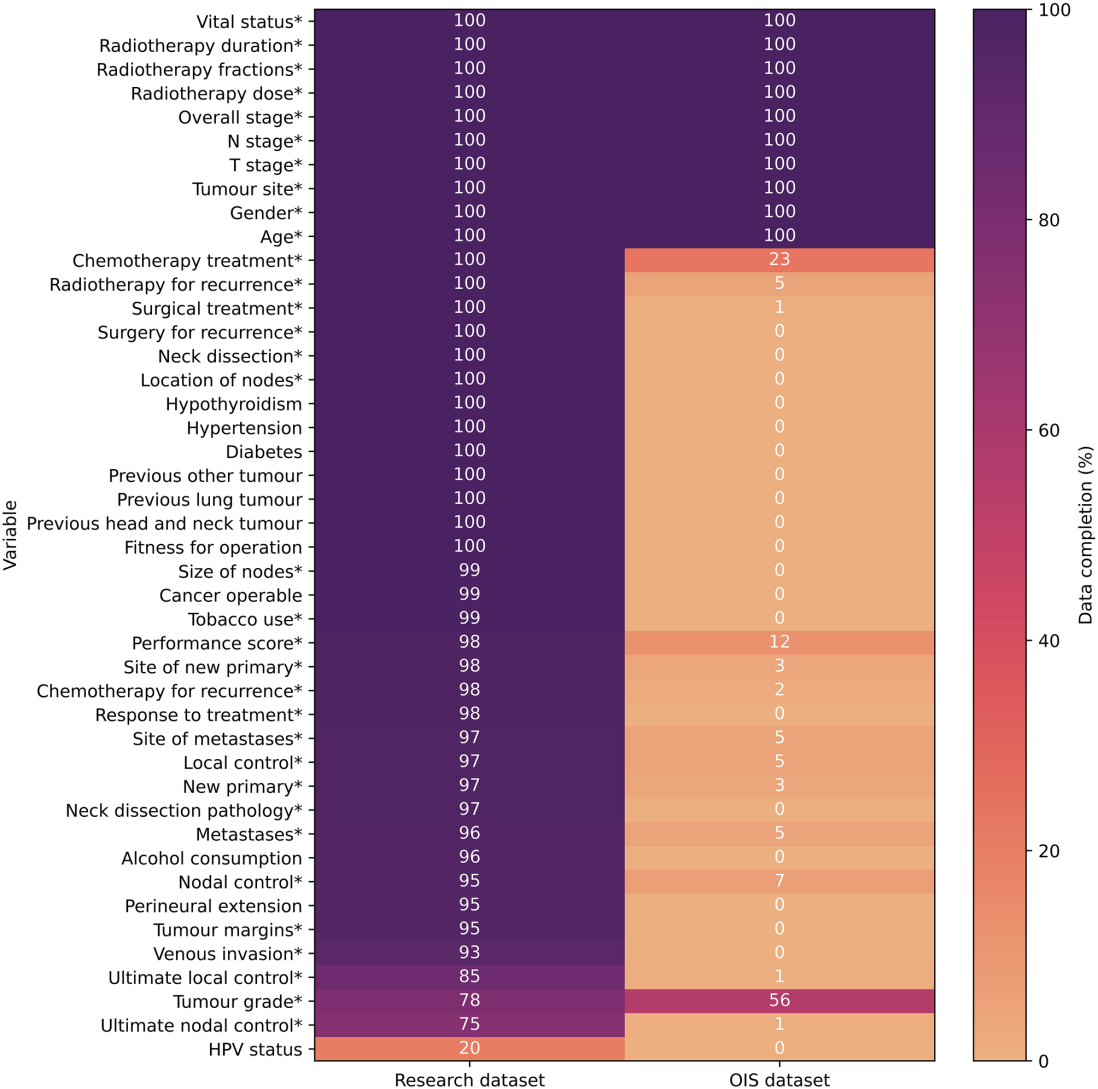
Heatmap representing the percent data completion in the research and OIS datasets, asterisks* indicates variables with available fields in the OIS. HPV; human papillomavirus

A total of 353 patients were eligible for inclusion in the study. Eighteen (5%) patients had two primary head and neck malignancies. All 44 variables that were investigated, and their level of completeness in both datasets, are displayed in Fig. 1, of which 13 could be statistically compared (Tables 1, 2 and 3). The overall data completion rate for the research and OIS datasets was 96% and 26% respectively for the 44 investigated variables, and 96% and 84% for the 13 analysed variables. The overall agreement between the two datasets for the 13 variables was 0.79.

**Table 1:** Patient characteristics (n = 353 patients)

	**Research dataset** **N (%)**	**OIS** **dataset** **N (%)**	**Percent complete** **N (%)**^a^	**Distribution p-value**^b^	**Agreement, SE, p-value**^c^
***Age, years, mean (SD)***	61.4 (11.0)	61.9 (11.1)	353 (100%)	**< 0.001**	0.993, 0.990–0.995, **< 0.001**
***Gender***					
Male	287 (81%)	287 (81%)	353 (100%)	1.000	1.000, < 0.001, **< 0.001**
Female	66 (19%)	66 (19%)			
***Performance (ECOG) status***					
0 – Normal	204 (58%)	31 (9%)	40 (12%)	NC	0.110, 0.136, 0.945
1 – Symptoms/self-care	127 (36%)	7 (2%)			
2 – Ambulatory < 50%	11 (3%)	0			
3 – Ambulatory > 50%	1 (< 1%)	2 (1%)			
4 – Bedridden	1 (< 1%)	0			
Unknown	9 (2%)	313 (88%)			

Bold indicates statistically significant *p*-value

*ECOG* Eastern Cooperative Oncology Group, *NC* not calculable, *SD* standard deviation, *SE* standard error

^**a**^Percent data completion for OIS dataset compared to the research dataset

^**b**^McNemar test for categorical data and paired t-test for normally distributed continuous data

^**c**^Cohen’s kappa test for categorical data, and intraclass correlation coefficient with 95% confidence interval for continuous data

**Table 2:** Tumour features (n = 353 patients)

	**Research dataset** **N (%)**	**OIS** **dataset** **N (%)**	**Percent complete** **N (%)**^a^	**Distribution p-value**^b^	**Agreement, SE, p-value**^c^
***Tumour site***					
Hypopharynx	18 (5%)	18 (5%)	353 (100%)	0.368	0.992, 0.006, **< 0.001**
Larynx	108 (30%)	108 (30%)			
Nasopharynx	16 (4%)	16 (4%)			
Oral cavity	63 (18%)	64 (18%)			
Oropharynx	144 (41%)	143 (41%)			
Paranasal sinus	2 (1%)	2 (1%)			
Salivary glands	2 (1%)	2 (1%)			
***Tumour grade***					
Well differentiated	32 (9%)	24 (7%)	196 (56%)	**< 0.001**	0.547, 0.033, **< 0.001**
Moderately well differentiated	152 (43%)	108 (30%)			
Poorly differentiated	91 (26%)	62 (18%)			
Undifferentiated	1 (< 1%)	2 (1%)			
Unknown	77 (22%)	157 (44%)			
***T stage (7***^***th***^ ***edition)***					
T1	115 (33%)	116 (33%)	353 (100%)	0.222	0.851, 0.023, **< 0.001**
T2	100 (28%)	101 (28%)			
T3	97 (27%)	102 (29%)			
T4	41 (12%)	34 (10%)			
***N stage (7***^***th***^ ***edition)***					
N0	171 (49%)	168 (48%)	353 (100%)	0.106	0.812, 0.027, **< 0.001**
N1	63 (18%)	62 (18%)			
N2	111 (31%)	118 (33%)			
N3	8 (2%)	5 (1%)			
***Overall stage (7***^***th***^ ***edition)***					
Stage I	70 (20%)	68 (19%)	353 (100%)	0.590	0.791, 0.027, **< 0.001**
Stage II	47 (13%)	43 (12%)			
Stage III	91 (26%)	96 (27%)			
Stage IV	145 (41%)	146 (42%)			

Bold indicates statistically significant *p*-value

*NC* not calculable, *SE* standard error

^**a**^Percent data completion for OIS dataset compared to the research dataset

^**b**^McNemar test for categorical data

^**c**^Cohen’s kappa test for categorical data

**Table 3:** Treatment and vital status (n = 353 patients)

	**Research dataset** **N (%)**	**OIS** **dataset** **N (%)**	**Percent complete** **N (%)**^a^	**Distribution p-value**^b^	**Agreement, SE, p-value**^c^
***RT dose, gray, median (IQR)***	64 (58–68)	66 (58–68)	353 (100%)	**0.018**	0.889, 0.862–0.910, **< 0.001**
***RT fractions, median (IQR)***	32 (28–34)	33 (28–34)	353 (100%)	**0.016**	0.856, 0.821–0.883, **< 0.001**
***RT duration, days, median (IQR)***	39 (36–41)	39 (36–42)	353 (100%)	0.972	0.818, 0.776–0.853, **< 0.001**
***Chemotherapy*** ^***d***^					
No	236 (67%)	16 (5%)	78 (23%)	0.625	0.871, 0.073, **< 0.001**
Yes	117 (33%)	62 (18%)			
Unknown	0	275 (77%)			
***Vital status***					
Alive	196 (56%)	231 (65%)	353 (100%)	**< 0.001**	0.689, 0.038, **< 0.001**
Dead	157 (44%)	122 (35%)			

Bold indicates statistically significant *p*-value

*HNC* head and neck cancer, *IQR* interquartile range, *NC* not calculable, *RT* radiotherapy, *SE* standard error

^**a**^Percent data completion for OIS dataset compared to the research dataset

^**b**^McNemar test for categorical data and Wilcoxon Signed Ranks test for non-normally distributed continuous data

^**c**^Cohen’s kappa test for categorical data, and intraclass correlation coefficient with 95% confidence interval for continuous data

^**d**^Cohen’s kappa compares the 68 and 58 patients treated by chemotherapy from 2012 in the research and OIS datasets respectively

### Patient characteristics

Three variables were statistically compared from the twelve that were investigated (Table 1). Data on age and gender were 100% complete in the OIS dataset, with perfect agreement for gender (81% male). The mean age was 61.4 years in the research dataset and 61.9 years in the OIS dataset, with high agreement (κ = 0.993, 95% CI 0.990–0.995, p < 0.001). The mean difference of 0.5 years was statistically significant (p < 0.001). Performance status was known for only 12% of the cohort in the OIS dataset compared to 98% in the research dataset (κ = 0.110, SE 0.136, p = 0.945).

### Tumour features

Table 2 displays five of the nine clinical tumour variables that could be compared. Tumour site was 100% complete in both datasets, demonstrating high agreement (κ = 0.992, SE 0.006, p < 0.001). Carcinoma of the oropharynx (41%) and larynx (30%) were the most common tumour sites. For stage, agreement was high for T stage (κ = 0.851, SE 0.023, p < 0.001) and N stage (κ = 0.812, SE 0.027, p < 0.001), and substantial for overall stage (κ = 0.791, SE 0.027, p < 0.001). In both datasets, almost half of all tumours were stage IV, reflected by the high incidence of N2/N3 stage disease. Data on grade was 78% and 56% complete in the research and OIS datasets, respectively, and agreement was moderate (κ = 0.547, SE 0.033, p < 0.001).

### Treatment details

Ten treatment factors were collected and four compared (Table 3). According to the research dataset, radiotherapy was delivered at a median dose of 64 Gy, a median of 32 fractions, over a median of 39 days. In the OIS dataset, radiotherapy was delivered at a median dose of 66 Gy, with 33 median fractions, over a median of 39 days. This resulted in high agreement for dose (κ = 0.889, 95% CI 0.862–0.910, p < 0.001), fractions (κ = 0.856, 95% CI 0.821–0.883, p < 0.001) and duration (κ = 0.818, 95% CI 0.776–0.853, p < 0.001). The median differences of two gray (p = 0.018) and one fraction (p = 0.016) were statistically significant.

Chemotherapy data was not routinely collected for diagnoses before 2012 in the OIS dataset, resulting in 22% completion compared to 100% for the research dataset. From 2012, 68 (19%) and 58 (16%) tumours were treated with chemotherapy according to the research and OIS datasets, respectively, resulting in high agreement (κ = 0.871, SE 0.073, p < 0.001). Only 1% of surgical data was available in the OIS dataset compared to 100% for the research dataset, preventing further analysis.

### Treatment outcomes

Information on treatment outcomes could not be compared since the number of tumours which responded to treatment could not be determined in the OIS dataset despite available fields (Fig. 1). Treatment outcome data was largely complete in the research dataset; treatment failed for 59 (17%) and 43 (12%) tumours at the local or nodal site, respectively. Subsequently, 27 and 18 retreated tumours experienced a second local and nodal failure, respectively. Thirty-two (9%) patients presented with metastasis during follow-up, and 51 (14%) patients with a new primary.

### Vital status

The date of last follow-up was only available in the research dataset, with a median follow-up of 3.9 years (IQR 1.7-7.0 years). The research dataset captured vital status and cause of death, enabling classification as HNC-related death, non-HNC-related death, or unknown cause, whereas the OIS dataset only included vital status (Table 3). Substantial agreement was observed for vital status (κ = 0.689, SE 0.038, p < 0.001). In the research dataset, 157 deaths were recorded, of which 81 (52%) were due to HNC, 73 (46%) from other causes, and 3 (2%) from unknown causes.

### Time expenditure

Data extraction and entry time was retrospectively estimated at 99 and 265 h for the OIS and research datasets, respectively. Query development and testing for the OIS dataset was estimated at 35 h with five hours of data curation.

## Discussion

Routinely collected HNC data sourced from a major metropolitan teaching and tertiary referral hospital OIS was less accurate and complete compared to a dataset compiled for research purposes. This led to varying levels of agreement when comparing the datasets, consequent of OIS data entry practices and utility. This is the first study to investigate the quality and completeness of routinely collected HNC data in an OIS.

Cancer centres rely on routinely collected patient information to inform decision-making and guide patient care; however, relatively few studies have examined the accuracy and completeness of OIS data. One US study investigated the concordance of OIS compared to cancer registry data for 11,110 patients [22]. The authors reported a high completion rate for data elements (age, race/ethnicity, gender, and smoking-related cancer) in the OIS, and an overall moderate agreement for these variables (κ = 0.78), comparable to the overall agreement in our study (κ = 0.79). Results from our study and that by LeLaurin et al. [22] support the conclusion that complete data does not imply high quality data.

Cancer centres collect data to suit their needs, including clinical, administrative, and reporting purposes. The OIS has the capacity to capture most of the clinically relevant demographic, diagnostic, treatment, and outcome data necessary for HNC prognostic research, however, not all user-defined fields are routinely completed. One study has demonstrated this, whereby two Australian hospitals utilised the same OIS for different purposes [21]. One hospital used the full capacity of the OIS administrative and clinical fields for documentation, while the other hospital used only the administrative fields. Our results indicate the OIS is used for both administrative and clinical purposes at this cancer centre; however, the collection of clinical information in the OIS is limited to the recording of clinical fields mandated for collection by the government, insufficient for research purposes.

OIS prescription fields (radiotherapy start and end date, dose, and fractions) are automatically populated following radiotherapy treatment, resulting in multiple doses for each patient, each signifying a treatment course, anatomical treatment field, or overlapping treatment fields [13]. Prior to analysis, OIS dose data was summarised as a singular total dose. In the clinical documents, radiation oncologists report the singular total dose delivered by the OIS. To determine whether radiotherapy data extracted from a single OIS using automated and manual methods resulted in the same data accuracy, a study evaluated 251 German meningioma patient records [28]. Automated extraction resulted in significantly lower retrieval time (35 h) and higher accuracy (93.9%) compared to manual processes (668 h, 91.2% accuracy, p = 0.009). In our study, extraction of radiotherapy data from the OIS was less accurate than manual extraction from clinical documents for the research dataset.

All other fields in the OIS require manual entry of information as structured or free-text. The information reported in structured fields such as stage and radiotherapy treatment were complete, though varied between the research and OIS datasets, while chemotherapy and surgical treatment information was limited in OIS dataset. One explanation is that staging data at the time of initial investigation may not have been updated in the OIS following subsequent investigation with more advanced diagnostic techniques. In the research dataset, a radiation oncologist retrospectively staged each patient, with no feedback mechanism in place to update the OIS. Although we observed high statistical agreement between the datasets for tumour stage, the agreement was inadequate for clinical or epidemiological research purposes, as TNM staging is a vital prognostic factor for HNC [5, 7, 9, 29]. Implementing feedback sessions between clinicians and data administrators may improve the quality of staging information and capture changes in disease stage over time [30].

Entry of chemotherapy and surgical treatment data in the OIS fields was poor, and largely complete in the research dataset. Free-text fields such as radiotherapy treatment site also resulted in variation between datasets. To improve the quality of radiotherapy data extracted from the OIS, standardisation of terminology is critical to ensure radiotherapy data is entered in a non-ambiguous manner for accurate data extraction and subsequent analysis [31–33]. Standardised treatment and outcome data are necessary to reliably investigate patterns of care and survival outcomes [5, 34, 35], and institutions contemplating research need to consider whether the structure of their OIS, data entry practices, staff and data availability, support their research needs.

Maintaining multiple datasets is neither practical nor cost-effective, an issue not unique to this OIS or cancer centre [36–38]. The goal is a single electronic health record that can serve multiple purposes, i.e., administration, clinical care, government reporting, and research. One approach is to improve data management practices without modifying the OIS. Establishing clinical leadership, commitment, and engagement with clerical, medical, and management staff across the department [21], with a strong collaborative information-sharing strategy and support for cultural change, are essential to identify, discuss, and implement processes to improve data management practices [39]. Successful implementation requires thorough planning, modification of training, and resource requirements, and regular auditing to optimise the utility of the OIS [39]. The data strategy should also detail the data standards (terminologies, vocabularies and coding schemes) and accountability, and include a clear vision of how the OIS will be used to support clinical practice and improve patient outcomes [21].

In conjunction with the above data strategy, another approach is to redesign the OIS or implement external customised systems to improve OIS clinical and research functionality. For example, instating mandatory completion of structured fields in the OIS improves data completeness [40]. Customised web-based electronic data capture systems can be used in tandem with an OIS to consolidate clinical information, reduce redundancy, and improve completeness of data fields, without detracting from clinical workflow, by reducing free-text data entry and increasing use of structured data fields [41].

A third approach is to consider data mining techniques using the clinical documents in the OIS, where certain fields can be anonymised prior to the use of these documents for medical research [42]. The research dataset demonstrates the required data are available in the clinical documentation saved within the OIS. Therefore, the application of natural language processing to categorise unstructured information from clinical documents into structured data is worthy of examination [43–46]. The availability of enriched clinical data may facilitate further research and collaboration and improve outcomes for people with HNC.

There were limitations to this study. The availability of data in the research dataset is dependent on clinicians reporting the information in the clinical documents. Multiple researchers were involved in the extraction of data for the research dataset, and differences in data interpretation may be present. Both datasets relied on manual data extraction, with the possibility of human error. Multisite comparisons to broadly understand OIS practices are not possible due to a lack of research datasets.

## Conclusion

Data manually extracted from unstructured clinical documents for research purposes was more complete and higher quality than data collected routinely in an OIS. The OIS dataset is not yet suitable for robust epidemiological research. Improved OIS data entry, integration with clinical activities and workflows, system usability, data dictionaries, and training are necessary before these data can be leveraged for robust research. Automated data mining techniques from electronic documents stored in the OIS should be investigated.

## Data Availability

Data are not available due to privacy/ethical restrictions.
